# Three-dimensional printing of complex biological structures by freeform reversible embedding of suspended hydrogels

**DOI:** 10.1126/sciadv.1500758

**Published:** 2015-10-23

**Authors:** Thomas J. Hinton, Quentin Jallerat, Rachelle N. Palchesko, Joon Hyung Park, Martin S. Grodzicki, Hao-Jan Shue, Mohamed H. Ramadan, Andrew R. Hudson, Adam W. Feinberg

**Affiliations:** 1 Department of Biomedical Engineering, Carnegie Mellon University, Pittsburgh, PA 15213, USA.; 2 Department of Chemistry, Carnegie Mellon University, Pittsburgh, PA 15213, USA.; 3 Department of Materials Science and Engineering, Carnegie Mellon University, Pittsburgh, PA 15213, USA.

**Keywords:** 3D printing, hydrogels, tissue engineering, alginate, collagen, fibrin, heart, perfusable vasculature, biomimetic

## Abstract

We demonstrate the additive manufacturing of complex three-dimensional (3D) biological structures using soft protein and polysaccharide hydrogels that are challenging or impossible to create using traditional fabrication approaches. These structures are built by embedding the printed hydrogel within a secondary hydrogel that serves as a temporary, thermoreversible, and biocompatible support. This process, termed freeform reversible embedding of suspended hydrogels, enables 3D printing of hydrated materials with an elastic modulus <500 kPa including alginate, collagen, and fibrin. Computer-aided design models of 3D optical, computed tomography, and magnetic resonance imaging data were 3D printed at a resolution of ~200 μm and at low cost by leveraging open-source hardware and software tools. Proof-of-concept structures based on femurs, branched coronary arteries, trabeculated embryonic hearts, and human brains were mechanically robust and recreated complex 3D internal and external anatomical architectures.

## Teaser

Freeform reversible embedding of suspended hydrogels enables three-dimensional printing of soft extracellular matrix biopolymers in biomimetic structures.

## INTRODUCTION

Over the past decade, the additive manufacturing (AM) of biomaterials has transitioned from a rapid prototyping tool used in research and development into a viable approach for the manufacturing of patient-specific medical devices. Key to this is the ability to precisely control structure and material properties in three dimensions and tailor these to unique anatomical and physiological criteria based on computed tomography (CT) and magnetic resonance imaging (MRI) medical imaging data. First-in-human applications include customized polyetherketoneketone bone plates for the repair of large cranial defects (*1*, *2*) and polycaprolactone bioresorbable tracheal splints for pediatric applications (*3*). The enabling three-dimensional (3D) printing technologies are primarily based on selective laser sintering of metal, ceramic, or thermoplastic microparticles; fused deposition modeling of thermoplastics, or on photopolymerization of photosensitive polymer resins (*4*, *5*), and have tremendous growth potential for surgical and medical devices (*4*, *6*) and scaffolds for tissue repair (*7*, *8*). However, these approaches are limited in their ability to 3D print very soft materials such as elastomers, gels, and hydrogels that are integral components of many medical devices and are required for most future applications in tissue engineering and regenerative medicine (*9*, *10*). Specifically, biological hydrogels composed of polysaccharides and/or proteins are a class of materials that are challenging to 3D print because they must first be gelled in situ during the fabrication process and then supported so that they do not collapse or deform under their own weight. Although the need for support materials is common across many AM techniques, it is particularly difficult for these soft biological hydrogels, where the elastic modulus is <100 kPa and there is a narrow range of thermal, mechanical, and chemical conditions that must be met to prevent damage to the materials and potentially integrated cells.

Current approaches for the 3D printing of biological hydrogels have achieved important advances but are still in need of significant improvement (*9*, *11*). For example, syringe-based extrusion has been used to 3D print polydimethylsiloxane (PDMS) elastomer and alginate hydrogel into multiple biological structures including the ear (*12*) and aortic heart valve (*13*, *14*). Other research teams have demonstrated the direct bioprinting of fibrin (*15*, *16*), gelatin (*17*), and mixtures of proteins derived from decellularized tissues (*18*) or cast extracellular matrix (ECM) gels around dissolvable templates (*19*). These results have expanded the range of materials that can be used and demonstrated the ability to incorporate and print live cells. There are also commercially available bioprinters from Organovo (*20*–*22*) and EnvisionTEC (*7*, *23*) that have expanded the accessibility of bioprinters beyond the groups that custom build their own systems. However, the complexity of microstructures and the 3D anisotropy that can be created remain limited; often, the structures printed are simple square lattices, similar to stacked Lincoln Logs, which do not recapitulate the microstructure of real tissues.

As a field, significant improvements are still needed in terms of the ability to directly manufacture using biologically relevant hydrogels, controlling microstructure and anisotropy in 3D, and expanding biological AM research by driving down the cost of entry while increasing the quality and fidelity of the printing process. Our goal was to specifically address five major challenges including (i) deposition and cross-linking of soft biomaterials and viscous fluids with elastic moduli of <100 kPa, (ii) supporting these soft structures as they are printed so that they do not collapse or deform, (iii) anisotropically depositing the material to match the microstructure of real tissue, (iv) removing any support material that is used, and (v) keeping cells alive during this whole process using aqueous environments that are pH-, ionic-, temperature-, and sterility-controlled within tight tolerances (*24*–*26*).

## RESULTS AND DISCUSSION

### Using a thermoreversible support bath to enable freeform reversible embedding of suspended hydrogels

**Fig. 1:**
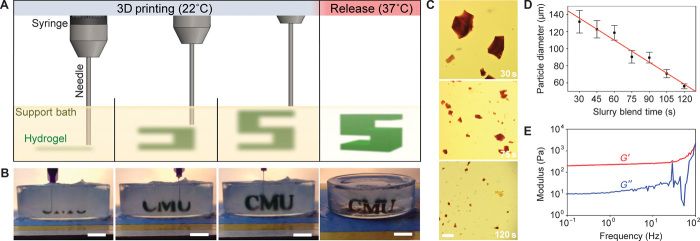
FRESH printing is performed by depositing a hydrogel precursor ink within the thermoreversible support bath consisting of gelatin microparticles and initiating gelling in situ through one of multiple cross-linking mechanisms. (**A**) A schematic of the FRESH process showing the hydrogel (green) being extruded and cross-linked within the gelatin slurry support bath (yellow). The 3D object is built layer by layer and, when completed, is released by heating to 37°C and melting the gelatin. (**B**) Images of the letters “CMU” FRESH printed in alginate in Times New Roman font (black) and released by melting the gelatin support (gray material in the petri dish). When the gelatin support melts the change in optical properties, convective currents and diffusion of black dye out of the alginate make it appear that the letters are deforming, although they are not. (**C**) Representative images of gelatin particles produced by blending for 30, 75, or 120 s. (**D**) The mean Feret diameter of gelatin particles as a function of blending time from 30 to 120 s (*n* > 1000 per time point; the red line is a linear fit and error bars indicate SD). (**E**) Rheological analysis of storage (*G*′) and loss (*G*″) modulus for gelatin support bath showing Bingham plastic behavior. Scale bars, 1 cm (B) and 1 mm (C).

Here, we report the development of a 3D bioprinting technique termed freeform reversible embedding of suspended hydrogels (FRESH). FRESH uses a thermoreversible support bath to enable deposition of hydrogels in complex, 3D biological structures and is implemented using open-source tools, serving as a highly adaptable and cost-effective biological AM platform. The key innovation in FRESH is deposition and embedding of the hydrogel(s) being printed within a second hydrogel support bath that maintains the intended structure during the print process and significantly improves print fidelity (Fig. 1, A and B, and movie S1). The support bath is composed of gelatin microparticles that act like a Bingham plastic during the print process, behaving as a rigid body at low shear stresses but flowing as a viscous fluid at higher shear stresses. This means that, as a needle-like nozzle moves through the bath, there is little mechanical resistance, yet the hydrogel being extruded out of the nozzle and deposited within the bath is held in place. Thus, soft materials that would collapse if printed in air are easily maintained in the intended 3D geometry. This is all done in a sterile, aqueous, buffered environment compatible with cells, which means cells can be extruded out of the printer nozzle with the hydrogel and maintain viability. Once the entire 3D structure is FRESH printed, the temperature is raised to a cell-friendly 37°C, causing the gelatin support bath to melt in a nondestructive manner. Although Wu *et al*. (*27*) previously described 3D printing of a hydrogel ink within a hydrogel support bath for omnidirectional printing, the fugitive ink was designed to leave microchannels within a permanent support bath that was ultravioletly cross-linked afterward to repair nozzle-induced damage. In contrast, FRESH enables the direct 3D printing of biologically relevant hydrogel inks including alginate, fibrin, collagen type I, and Matrigel within a fugitive support bath designed to be removed afterward.

FRESH is implemented on a MakerBot Replicator modified with a custom syringe-based extruder designed for precision hydrogel deposition. All plastic parts to convert the MakerBot into a bioprinter are printed in polylactic acid (PLA) using the stock thermoplastic extruder, which is then replaced with the custom syringe-based extruder [the STL (stereolithography) file can be downloaded from http://3dprint.nih.gov/]. Our syringe-based extruder uses the stepper motor, taken from the original extruder, to move the plunger of a 3-ml syringe via a direct gear drive (fig. S1). The overall size and mass is comparable to the original extruder and, once mounted, integrates seamlessly with the MakerBot hardware and software, requiring only calibration of the number of motor steps that extrudes a given volume of fluid. Typically, we use a 150-μm-diameter stainless steel needle on the end of the syringe, but a range of needle diameters can be selected to control the volume of material being extruded.

The FRESH support bath consists of a slurry of gelatin microparticles processed to have a Bingham plastic rheology. To do this, we blended a solid block of gelatin hydrogel to break up the material into microparticles and then centrifuged it to remove the supernatant and produce the final slurry (fig. S2). Increasing the blending time decreases microparticle size (Fig. 1C), with a blending time of 120 s producing microparticles with a mean Feret diameter of 55.3 ± 2 μm (Fig. 1D). Rheometry confirmed that the gelatin slurry that was blended for 120 s behaved like a Bingham plastic (Fig. 1E), not yielding until a threshold shear force is reached. Maintaining the gelatin slurry at room temperature (~22°C) preserves these rheological properties. For FRESH, the gelatin support slurry is loaded into a container of sufficient size to hold the part to be printed. In addition to its rheological and thermoreversible properties, gelatin was selected as the support bath material because it is biocompatible (*28*, *29*). This is important, as it is unlikely that 100% of the gelatin is removed during the release process because it is a denatured form of collagen type I that can self-associate and bind to polysaccharides and other ECM proteins such as fibronectin (*30*, *31*). Thus, it is unlikely that any small amount of residual gelatin will negatively affect cell integration and may actually enhance adhesion through integrin binding (*32*).

### Characterization of 3D printed hydrogels using FRESH

**Fig. 2:**
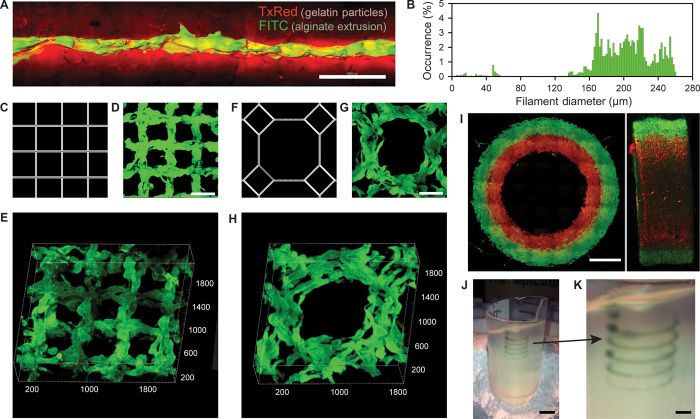
Analysis of the hydrogel filaments and structures fabricated using FRESH. (**A**) A representative alginate filament (green) embedded within the gelatin slurry support bath (red). (**B**) Histogram of the diameter of isolated alginate filaments within the gelatin support bath showing a range from 160 to 260 μm. (**C** to **E**) A standard square lattice pattern commonly used for infill in 3D printing FRESH printed in fluorescent alginate (green) and viewed (D) top down and (E) in 3D. (**F** to **H**) An octagonal infill pattern FRESH printed in fluorescent alginate (green) and viewed (G) top down and (H) in 3D. (**I**) Example of a two-material print of coaxial cylinders in red and green fluorescently labeled alginate with a continuous interface shown in top down and lateral cross sections. (**J**) An example of a freeform, nonplanar FRESH print of a helix shown embedded in the gelatin support bath. (**K**) A zoomed-in view of the helix demonstrating that FRESH can print in true freeform and is not limited to standard layer-by-layer planar fabrication. Scale bars, 1 mm (A), 500 μm (D and G), 2 mm (I), 10 mm (J), and 2.5 mm (K).

FRESH works by extruding the liquid phase material from the syringe into the support bath, where the material must rapidly gel into a filament without diffusing away. This gelation process occurs via rapid cross-linking of the polymer molecules into a network, and the cross-linking mechanism depends on the hydrogel being 3D printed. We have validated this process using fluorescently labeled alginate cross-linked by divalent cations (0.16% CaCl_2_) added to the support bath. A representative alginate filament embedded in the support bath illustrates that the gelatin microparticles are moved out of the way but still influence the surface morphology of the filament (Fig. 2A). As the alginate gels, there are visible “spurs” that form in between microparticles. However, these are not necessarily a problem in the context of a larger 3D printed structure because filaments fuse together to form the 3D printed part and thus these spurs may actually enhance this process by better bridging filaments. For this representative filament, the diameter of the extrusion was 199 ± 41 μm (Fig. 2B). However, the diameter of the extruded hydrogel filament depends on a large number of factors including the hydrogel being printed and its cross-linking kinetics, gelatin microparticle size, nozzle diameter, extruder translation speed, and flow rate. Thus, similar to 3D printing of most materials, the resolution and morphology of a print depend on a number of machine settings and require optimization for each material used.

Although the properties of single filaments are important, it is the ability of filaments to fuse into larger-scale structures that is required for 3D printing. Metal and plastic 3D printing typically produces parts that are <100% solid, creating an external skin that is infilled using a repeating geometric structure with a defined porosity. For FRESH, we used rectilinear and octagonal infill algorithms to generate patterns of interconnected alginate filaments (Fig. 2, C to H). The rectilinear infill is a simple square lattice structure (Fig. 2C) that we FRESH printed at a 500-μm pitch (Fig. 2D). Confocal imaging and 3D rendering demonstrate that there is interconnectivity between filaments in the *x*, *y*, and *z* axes (Fig. 2E). The octagonal infill is a more complex pattern of squares and octagons (Fig. 2F) that we FRESH printed at a 750-μm pitch (Fig. 2G). A 3D rendering again demonstrates the interconnectivity between filaments in the *x*, *y*, and *z* axes (Fig. 2H). It should be noted that the fidelity of these infill patterns is comparable to that achieved using the stock thermoplastic extruder to print the same geometries in PLA, and further improvements are anticipated by performing FRESH on better hardware with optimized print parameters.

FRESH can also be used to 3D print complex multimaterial parts and in nonplanar geometries. Dual syringe-based extruders can be mounted onto the MakerBot (fig. S1) and directly leverage the dual-extruder printing capability built into the software to alternate between extruders (movie S2). To demonstrate dual-material printing, we printed two different fluorescently labeled alginates in concentric cylinders. Multiphoton imaging shows distinct layers, each 1 mm wide, integrated together throughout a 3-mm thickness (Fig. 2I). Uniquely, FRESH is also not limited to standard layer-by-layer 3D printing and can freeform deposit material in 3D space with high fidelity as long as the extruder does not pass through previously deposited material. This is demonstrated by printing a single filament along a helical path (Fig. 2, J and K, and movie S3). This is a continuous, single filament with the extruder simultaneously moving in *x*, *y*, and *z*, showing the ability to deposit material in highly anisotropic structures in all three axes.

### 3D printing of complex biological structures

**Fig. 3:**
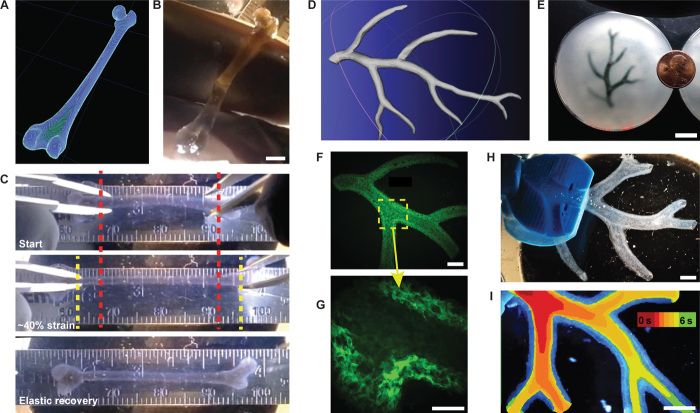
FRESH printing of biological structures based on 3D imaging data and functional analysis of the printed parts. (**A**) A model of a human femur from 3D CT imaging data is scaled down and processed into machine code for FRESH printing. (**B**) The femur is FRESH printed in alginate, and after removal from the support bath, it closely resembles the model and is easily handled. (**C**) Uniaxial tensile testing of the printed femur demonstrates the ability to be strained up to 40% and elastically recover. (**D**) A model of a section of a human right coronary arterial tree from 3D MRI is processed at full scale into machine code for FRESH printing. (**E**) An example of the arterial tree printed in alginate (black) and embedded in the gelatin slurry support bath. (**F**) A section of the arterial trees printed in fluorescent alginate (green) and imaged in 3D to show the hollow lumen and multiple bifurcations. (**G**) A zoomed-in view of the arterial tree shows the defined vessel wall that is <1 mm thick and the well-formed lumen. (**H**) A dark-field image of the arterial tree mounted in a perfusion fixture to position a syringe in the root of the tree. (**I**) A time-lapse image of black dye perfused through the arterial tree false-colored at time points of 0 to 6 s to show flow through the lumen and not through the vessel wall. Scale bars, 4 mm (B), 10 mm (E), 2.5 mm (F), 1 mm (G), and 2.5 mm (H and I).

FRESH was next used to print complex biological structures based on medical imaging data to demonstrate its capability to fabricate complex geometries. Further, we wanted to validate that prints were mechanically robust and could be formed from multiple types of protein and polysaccharide hydrogels. First, a human femur from CT data (Fig. 3A) was scaled down to a length of ~35 mm and a minimum diameter of ~2 mm and FRESH printed in alginate (Fig. 3B). The 3D printed femur only mimicked the external structure (surface) of the real femur and had a solid infill. Applying uniaxial strain showed that the femur could undergo ~40% strain and recover elastically (Fig. 3C and movie S4), validating that there was mechanical fusion between the printed alginate layers. Further, the femur could be bent in half and elastically recover and, when strained to failure, fractured at an oblique angle to the long axis of the bone, confirming that failure was not due to layer delamination (movie S5). Next, we created a simple bifurcated tube in CAD (computer-aided design) to demonstrate the ability to FRESH print a hollow structure (fig. S3A). We used both the femur and bifurcated tube to show that other ECM hydrogels including collagen type I and fibrin can be FRESH printed with comparable fidelity to alginate (fig. S3, B to D, and movie S6). Printing multiple copies of the same bifurcated tube continuously for 4 hours also confirmed that the platform was thermally stable and that support bath rheological properties did not change over this period (fig. S3, C and D). Further, sheets of C2C12 myoblasts suspended in a mixture of fibrinogen, collagen type I, and Matrigel were printed at 20°C under sterile conditions and showed 99.7% viability by LIVE/DEAD staining (fig. S4, A and B). Multiday studies using C2C12 myoblasts and MC3T3 fibroblasts showed that cells were well distributed in 3D (fig. S4, C and E, respectively) and, over a 7-day culture period, formed a high-density cellular network (fig. S4, D and F, respectively). These examples demonstrate that FRESH can 3D print mechanically robust parts with biomimetic structure (Fig. 3C) and high repeatability (fig. S3, C and D) from a range of ECM hydrogels including collagen, fibrin, and Matrigel (figs. S3 and S4) and with embedded cells (fig. S4).

Additional mechanical characterization was performed by creating cast and 3D printed alginate dog bones (fig. S5A) and subjecting them to uniaxial tensile testing to generate stress-strain curves (fig. S5B), with the linear region from 5 to 20% strain used to calculate the elastic modulus (fig. S5C). Alginate is widely used in the tissue engineering field, and our results were comparable to those previously reported (*33*), although our gels were stiffer because of higher alginate and calcium concentrations. The cast alginate had a strain-to-failure of 42 ± 8% (fig. S5D), about two times that of the 3D printed alginate, and an elastic modulus of 446 ± 72 kPa (fig. S5E), about nine times that of the 3D printed alginate. Part of this difference is because the 3D printed alginate dog bones were printed with 50% infill, effectively reducing the true cross-sectional area and introducing internal voids that initiated cracks at lower strains. Normalizing for the 50% infill by taking the cross-sectional area as half of that measured externally increased the elastic modulus from 51 ± 14 kPa to 102 ± 27 kPa, which is ~25% of the cast alginate modulus. The lower mechanical properties of the 3D printed alginate were expected because the layer-by-layer fabrication approach is known to impart defects and material anisotropy (*34*, *35*). However, these results, in combination with the straining of the 3D printed femur, demonstrate the mechanical fusion between printed layers and show that FRESH can be used to fabricate soft structures with mechanical integrity.

We next evaluated the ability to fabricate a more complex, perfusable structure using MRI data of part of the right coronary artery vascular tree and creating a hollow lumen with a wall thickness of <1 mm (Fig. 3D) (*36*). This was FRESH printed to scale with an overall length from trunk to tip of ~4.5 cm and contained multiple bifurcations with 3D tortuosity (Fig. 3E and movie S7). Arterial trees printed using fluorescent alginate confirmed that the internal lumens and bifurcations were well formed (Fig. 3F) and that a wall thickness of <1 mm and lumen diameters of 1 to 3 mm were achieved (Fig. 3G). Detailed structural analysis comparing the 3D model (fig. S6A) to the 3D printed arterial tree (fig. S6B) showed good fidelity and accurate anatomical structure with <15% variation in overall length and width and angles of the major bifurcations within ≤3°. Analysis of the wall thickness and lumen diameter confirmed that the 3D model (fig. S6C) was comparable to the 3D printed arterial tree (fig. S6D), although the printed wall thickness was increased and the lumen diameter decreased to ensure mechanical integrity of the overall vessel network for perfusion studies. A custom fixture to hold the arterial tree was 3D printed in PLA (Fig. 3H and fig. S7) and used to perfuse the print. Black dye pumped through the arterial tree confirmed that it was patent and manifold and that hydrogel density was sufficient to prevent diffusion through the wall (Fig. 3I and movie S8). Similar to the mechanical testing of the femur (Fig. 3C) and dog bones (fig. S5), the minimal diffusion through the arterial wall confirmed that the alginate layers were well fused together, forming a solid structure.

**Fig. 4:**
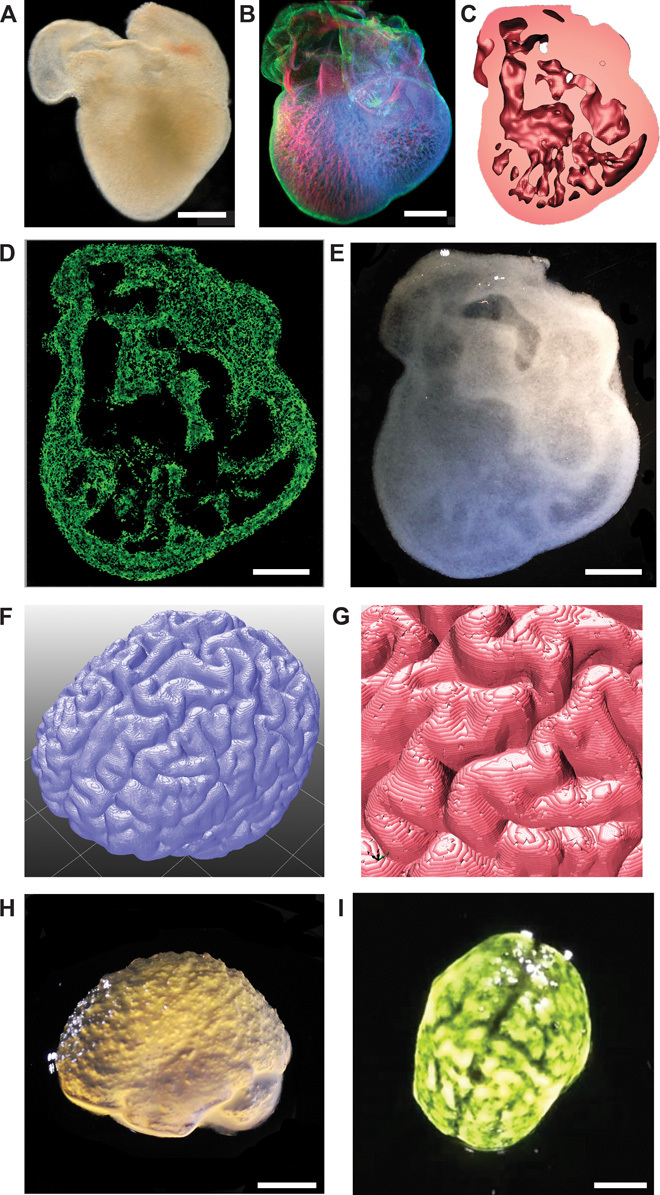
FRESH printed scaffolds with complex internal and external architectures based on 3D imaging data from whole organs. (**A**) A dark-field image of an explanted embryonic chick heart. (**B**) A 3D image of the 5-day-old embryonic chick heart stained for fibronectin (green), nuclei (blue), and F-actin (red) and imaged with a confocal microscope. (**C**) A cross section of the 3D CAD model of the embryonic heart with complex internal trabeculation based on the confocal imaging data. (**D**) A cross section of the 3D printed heart in fluorescent alginate (green) showing recreation of the internal trabecular structure from the CAD model. The heart has been scaled up by a factor of 10 to match the resolution of the printer. (**E**) A dark-field image of the 3D printed heart with internal structure visible through the translucent heart wall. (**F**) A 3D rendering of a human brain from MRI data processed for FRESH printing. (**G**) A zoomed-in view of the 3D brain model showing the complex, external architecture of the white matter folds. (**H**) A lateral view of the brain 3D printed in alginate showing major anatomical features including the cortex and cerebellum. The brain has been scaled down to ~3 mm in length to reduce printing time and test the resolution limits of the printer. (**I**) A top down view of the 3D printed brain with black dye dripped on top to help visualize the white matter folds printed in high fidelity. Scale bars, 1 mm (A and B) and 1 cm (D, E, H, and I).

Finally, we evaluated the ability to FRESH print 3D biological structures with complex internal and external architectures that would be extremely challenging or impossible to create using traditional fabrication techniques. First, we selected a day 5 embryonic chick heart (Fig. 4A) because of the complex internal trabeculations. We fixed and stained the heart for cell nuclei, F-actin, and fibronectin and generated a 3D optical image using confocal microscopy (Fig. 4B). The 3D optical image was then thresholded, segmented, and converted into a solid model for 3D printing (Fig. 4C and fig. S8). The diameter of the actual embryonic heart (~2.5 mm) was scaled up by an order of magnitude (~2.5 cm) to better match the resolution of the printer and FRESH printed using fluorescently labeled alginate. The printed heart was then imaged using a multiphoton microscope to generate a cross section through the structure (Fig. 4D) showing internal trabeculation comparable to that in the model (Fig. 4C). Comparing the 3D model, G-code machine path, and final printed alginate heart (fig. S9, A to C) showed good co-registration of primary features when overlaid on one another (fig. S9, D to F). A dark-field image of the whole 3D printed heart provided further validation of print fidelity and the ability to fabricate complex internal structures down to the submillimeter length scale (Fig. 4E). Dimensional analysis comparing the 3D heart model (fig. S9G) to the 3D printed heart (fig. S9H) demonstrated nearly identical length, width, and size of major internal structures with <10% variability. Overlaying images of the 3D model and printed heart helped further visualize the co-registration of the internal trabeculations and other anatomical features (fig. S9I). This embryonic heart is a good example of the types of structures that can be 3D printed with FRESH but are not possible to fabricate using traditional approaches because of the complex internal architecture.

To create complex external surface structures, we used an MRI image of the human brain (Fig. 4F) because of the intricate folds in the cortical tissues. A high-resolution view of the 3D brain model shows the surface in detail (Fig. 4G); however, the internal structure of the brain was solid infill. The embryonic heart model was scaled up in size, whereas the human brain model was scaled down to 3 cm in length to evaluate the resolution limits of the printer and reduce print times. The model of the exterior surface of the human brain was 3D printed using alginate, and different regions including the frontal and temporal lobes of the cortex and the cerebellum were well defined (Fig. 4H). Visualization of the brain surface was enhanced with black dye and revealed structures corresponding to the major folds of the cerebral cortex in the 3D model (Fig. 4I and movie S9). A more detailed comparison confirmed the similar morphology of multiple surface folds of the cerebral cortex between the 3D model and the 3D printed brain (fig. S10). Together, both the 3D printed embryonic heart and brain demonstrate the unique ability of FRESH to print hydrogels with complex internal and external structures.

Looking forward, can we leverage these FRESH bioprinting capabilities to engineer soft hydrogel scaffolds for advanced tissue engineering applications? In terms of complex scaffold design, our results demonstrate the ability to fabricate a wide range of 3D biological structures based on 3D imaging data with spatial resolution and fidelity that match or exceed previous results. Further, this is directly done with natural biopolymers such as alginate, fibrin, and collagen type I, which are cross-linked by ionic, enzymatic, and pH/thermally driven mechanisms, respectively. This flexibility in materials used and architectures printed defines a new level of capability for the AM of soft materials. The square and octagonal infill patterns (Fig. 2, C to H) show results comparable to those achieved with thermoplastics (for example, PLA) printed on the stock MakerBot Replicator printer we used, suggesting that we may be limited by the hardware. We anticipate that higher resolution is possible using higher-precision printers, smaller-diameter needles, and gelatin slurries with a smaller particle diameter. Cost is also an important consideration for the future expansion of 3D bioprinting as a tissue biofabrication platform, as commercially available and custom-built printers currently cost more than $100,000 and/or require specialized expertise to operate (*7*, *17*, *20*–*23*, *27*). In contrast, FRESH is built on open-source hardware and software and the gelatin slurry is low cost and readily processed using consumer blenders. To emphasize the accessibility of the technology, we implemented FRESH on a $400 3D printer (Printrbot Jr, movie S10) and the STL file to 3D print the custom syringe-based extruder can be downloaded from http://3dprint.nih.gov/. It should be acknowledged that the direct bioprinting of functional tissues and organs requires further research and development to become fully realized, and a number of companies and academic laboratories are actively working toward this goal. The low cost of FRESH and the ability to 3D print a range of hydrogels should enable the expansion of bioprinting into many academic and commercial laboratory settings and accelerate important breakthroughs in tissue engineering for a wide range of applications, from pharmaceutical testing to regenerative therapies.

## MATERIALS AND METHODS

### Modification of a MakerBot Replicator for syringe-based extrusion

All 3D printing was performed using a MakerBot Replicator (MakerBot Industries) modified with a syringe-based extruder (fig. S1A). To do this, we removed the stock thermoplastic extruder assembly from the plastic *x*-axis carriage and replaced it with a custom-built syringe pump extruder (fig. S1, B and C). The syringe pump extruder was designed to use the NEMA-17 stepper motor from the original MakerBot thermoplastic extruder and mount directly in place of the extruder on the *x*-axis carriage. The syringe pump extruder was printed in acrylonitrile butadiene styrene and PLA plastic using the thermoplastic extruder on the MakerBot before its removal. By using the same stepper motor, the syringe pump extruder was natively supported by the software that came with the printer. The design for the syringe pump extruder can be downloaded as an STL file from http://3dprint.nih.gov/ that can be printed on any RepRap or MakerBot 3D printer. In addition to a single extruder configuration, multiple syringe pump extruders could be mounted in a dual-extruder configuration, enabling 3D printing of multiple materials at one time (fig. S1D). No software modifications were necessary to operate the printer in single- or dual-extruder modes, aside from settings corresponding to nozzle diameter, filament diameter, and “start/end” G-code found in the software responsible for controlling the 3D printer.

### Preparation and analysis of gelatin slurry support bath

To create the gelatin slurry support bath, we mixed 150 ml of 4.5% (w/v) gelatin (Type A, Thermo Fisher Scientific) in 11 mM CaCl_2_ (Sigma-Aldrich) into a solution and then gelled it for 12 hours at 4°C in a 500-ml mason jar (Ball Inc.). Next, 350 ml of 11 mM CaCl_2_ at 4°C was added to the jar and its contents were blended (at “pulse” speed) for a period of 30 to 120 s on a consumer-grade blender (Osterizer MFG) (fig. S2A). Then, the blended gelatin slurry was loaded into 50-ml conical tubes (fig. S2B) and centrifuged at 4200 rpm for 2 min, causing slurry particles to settle out of suspension (fig. S2C). The supernatant was removed and replaced with 11 mM CaCl_2_ at 4°C. The slurry was vortexed back into suspension and centrifuged again. This process was repeated until no bubbles were observed at the top of the supernatant, which indicated that most of the soluble gelatin was removed. At this point, gelatin slurries could be stored at 4°C. For FRESH printing, the slurry was poured into a petri dish or a container large enough to hold the object to be printed (fig. S2D). Any excess fluid was removed from the gelatin slurry support bath using Kimwipes (Kimberly-Clark), which produced a slurry material that behaved like a Bingham plastic. All 3D printing was performed using gelatin blended for 120 s.

To measure the effect of blend time on gelatin particle size, we blended the gelatin for periods of 30, 45, 60, 75, 90, 105, and 120 s. Blend times longer than 120 s were not used because the gelatin particles began to entirely dissolve into the solution. For each blend time analyzed, 500 μl of slurry was removed and diluted to 10 ml with 11 mM CaCl_2_ and 0.1% (w/v) black food coloring (McCormick & Co.). Then, 140 μl of each diluted sample was mounted on a coverslip and imaged with a digital camera (D7000 SLR, Nikon) mounted on a stereomicroscope with oblique illumination (SMZ1000, Nikon). For each image, ImageJ (National Institutes of Health) (*37*) was used to enhance contrast, convert to LAB color space, and apply a lightness threshold. ImageJ was then used to count particles and measure their Feret diameters, areas, and circumferences using the “analyze particle” function. Linear regression of particle diameter as a function of time was performed using SigmaPlot 11 (Systat Software Inc.).

To measure the rheological properties of the gelatin slurry support bath, we blended the gelatin for 120 s and then prepared it as described for the FRESH 3D printing process. The slurry was loaded onto a Gemini 200 Rheometer with a 40-mm, 4° cone (Malvern) and analyzed in frequency sweep from 0.001 to 100 Hz at 150-μm separation and 25°C. The storage (*G*′) and loss (*G*″) moduli were measured and recorded in Microsoft Excel and plotted using SigmaPlot 11.

### Preparation of hydrogel inks for 3D printing

A solution of 2.0% (w/v) sodium alginate (FMC BioPolymer), 0.02% (w/v) 6-aminofluorescein [fluorescein isothiocyanate (FITC), Sigma], 0.022% (w/v) 1-ethyl-3-(3dimethylaminopropyl)carbodiimide (Sigma), and 0.025% (w/v) sulfo-*N*-hydroxysuccinimide (Sigma) in distilled water was prepared and stirred for 48 hours at 20°C to prepare fluorescently labeled alginate for 3D printing. Unreacted FITC was removed from FITC-labeled alginate by five consecutive 12-hour dialysis shifts against 2% (w/v) sodium alginate at 4°C in dialysis cassettes (Slide-A-Lyzer 3.5k MWCO, Thermo Fisher). After dialysis, 100 μl of FITC-labeled alginate was added to a 10-ml solution of 4% (w/v) sodium alginate, 0.4% (w/v) hyaluronic acid (Sigma), and 0.1% (w/v) black food coloring (for visualization during printing) to create a fluorescently labeled alginate ink. Fluorescent alginate prints were imaged using a Leica SP5 multiphoton microscope with a 10× [numerical aperture (NA) = 0.4] objective and a 25× (NA = 0.95) water immersion objective. Higher-magnification images were obtained using a Zeiss LSM 700 confocal microscope with a 63× (NA = 1.4) oil immersion objective. Bimaterial prints and arterial tree prints were imaged using a Nikon AZ-C2 macro confocal microscope with a 1× (NA = 0.1) objective. 3D image stacks were deconvolved with AutoQuant X3 and processed with Imaris 7.5 (Bitplane Inc.).

To prepare fibrinogen for 3D printing of fibrin constructs, we prepared a solution of fibrinogen (10 mg/ml; VWR), 0.5% (w/v) hyaluronic acid (Sigma), 1% (w/v) bovine serum albumin (Sigma), 10 mM sodium HEPES (Sigma), and 1× phosphate-buffered saline (PBS; VWR) and loaded it into a syringe for printing. To ensure cross-linking of the fibrinogen into fibrin once printed in the support bath, we supplemented the baths with thrombin (0.1 U/ml; VWR). Fibrin prints were released from the bath material by incubation at 37°C for at least 1 hour (fig. S3C).

For 3D printing of collagen, rat tail collagen type I (BD Biosciences) at concentrations ranging from 8.94 to 9.64 mg/ml in 0.02 N acetic acid was used as received without further modification. To ensure cross-linking of collagen into a gel after extrusion, the support bath was supplemented with 10 mM HEPES to maintain a pH of ~7.4 and neutralize the acetic acid. After printing, scaffolds were incubated at 37°C for at least 1 hour to further cross-link the collagen (fig. S3D) and melt the support bath.

For 3D printing of cellularized constructs, components of a multicomponent ECM ink were prepared at 4°C under sterile conditions in a biosafety cabinet. The ECM ink consisted of a solution of collagen type I (2 mg/ml; BD Biosciences), Matrigel (0.25 mg/ml; BD Biosciences), fibrinogen (10 mg/ml; VWR), 0.5% (w/v) hyaluronic acid, 1% (w/v) bovine serum albumin (Sigma), 10 mM sodium HEPES (Sigma), and 1× PBS (VWR), which was prepared and thoroughly mixed at 4°C. This specific protein and polysaccharide mixture was experimentally determined to quickly gel while maintaining viability of printed cells. C2C12 myoblasts or MC3T3-E1.4 cells were suspended in media at a concentration of 8 × 10^6^ cells/ml and diluted 1:4 with the ECM mixture to create a final concentration of 2 × 10^6^ cells/ml. The cellularized ink was then loaded into a sterile syringe used in the 3D printer. To ensure cross-linking of the ECM-based ink once printed, we supplemented the support bath with 10 mM HEPES and thrombin (0.1 U/ml).

### The FRESH 3D printing process

Digital 3D models for FRESH prints were created using 3D imaging data or designed using SolidWorks software (Dassault Systèmes). The files for the human femur and coronary artery tree were downloaded from the BodyParts3D database (*36*). The model of the human brain was provided under creative commons licensing by A. Millns (Inition Co.). The 3D digital models were opened in MeshLab (http://meshlab.sourceforge.net/) to be exported in the STL file format. For the 3D model of the coronary artery tree, only the outer surface was provided by the BodyParts3D database; hence, the arterial tree was resampled to create a smaller daughter surface with inverted normals. When both surfaces were combined, a hollow model with internal and external surfaces with a wall thickness of ~1 mm resulted, which was exported as an STL file for printing. All STL files were processed by Skeinforge (http://fabmetheus.crsndoo.com/) or KISSlicer (www.kisslicer.com/) software and sliced into 80-μm-thick layers to generate G-code instructions for the 3D printer. G-code instruction sets were sent to the printer using ReplicatorG (http://replicat.org/), an open-source 3D printer host program.

Hydrogel precursor inks were first drawn into a 2.5-ml syringe (Model 1001 Gastight Syringe, Hamilton Company) with a 150-μm-ID (inside diameter), 0.5-inch stainless steel deposition tip needle (McMaster-Carr) used as the nozzle to perform FRESH printing. The syringe was then mounted into the syringe pump extruder on the 3D printer (fig. S1, B and C). A petri dish or similar container large enough to hold the part to be printed was filled with the gelatin slurry support bath and manually placed on the build platform, and the container was held in place using a thin layer of silicone grease. The tip of the syringe needle was positioned at the center of the support bath in *x* and *y* and near the bottom of the bath in *z* before executing the G-code instructions. It is important to initiate FRESH 3D printing within 30 s of placing the syringe extruder in the support bath to avoid excessive cross-linking of material and clogging in the nozzle. Scaffolds were printed in a temperature-controlled room at 22 ± 1°C over a period of 1 min to 4 hours depending on the size and complexity of the printed construct as well as the ink used. For cellularized constructs, sterility was maintained by printing in a biosafety cabinet. Embedded constructs were heated to 37°C directly on the printer’s platform, placed on a dry bath, or placed inside an incubator to liquefy the support bath and release a print after FRESH. Once the gelatin was melted, alginate prints were rinsed with 11 mM CaCl_2_ and stored at 4°C. Once the gelatin was melted for collagen and fibrin prints, the objects were rinsed with 1× PBS and stored at 4°C. For multicomponent ECM prints containing cells, scaffolds were rinsed with the appropriate culture medium based on the incorporated cell types and incubated at 37°C.

### Cell culture and fluorescent staining

All reagents were purchased from Life Technologies unless otherwise specified. The MC3T3-E1.4 fibroblast cell line and prints containing MC3T3 cells [CRL-2593, American Type Culture Collection (ATCC)] were cultured in α-MEM (minimum essential medium) supplemented with 10% fetal bovine serum (FBS; Gibco Labs), penicillin (100 U/ml), and streptomycin (100 μg/ml). The C2C12 myoblast cell line and prints containing C2C12 cells (CRL-1722, ATCC) were cultured at 37°C under 5% CO_2_ in Dulbecco’s modified Eagle’s medium supplemented with 10% (v/v) FBS, 1% (v/v) l-glutamine (200 mM), penicillin (100 U/ml), and streptomycin (100 μg/ml), based on published methods (*38*).

Cell viability after FRESH printing was assessed by performing a LIVE/DEAD assay (Life Technologies) on prints containing C2C12 cells (fig. S4, A and B). Each print was first washed with Opti-MEM media containing 2% FBS and 2% 10,000-U penicillin-streptomycin solution and incubated at 37°C under 5% CO_2_ for 30 min. The prints were then removed from the incubator, rinsed with 1× PBS, incubated in 2 ml of PBS with 2 μl of calcein AM and 4 μl of ethidium homodimer per sample for 30 min, and then imaged on a Zeiss LSM 700 confocal microscope. The number of live and dead cells in each of the five images per three independent samples was counted and the percent viability was calculated by dividing the number of live cells by the number of total cells per image.

Prints containing cells were cultured for up to 7 days and analyzed at 1- and 7-day time points to verify cell survival and growth. After 1 and 7 days of culture, printed sheets were rinsed with 1× PBS (supplemented with 0.625 mM MgCl_2_ and 0.109 mM CaCl_2_) at 37°C, fixed in 4% (w/v) formaldehyde (Polysciences Inc.) for 15 min, and then washed three times in 1× PBS. Fixed prints were incubated for 12 hours in a 1:200 dilution of 4′,6-diamidino-2-phenylindole (DAPI; Life Technologies) and a 3:200 dilution of phalloidin conjugated to Alexa Flour 488 (Life Technologies). Prints were then washed three times in PBS and mounted with ProLong Gold antifade reagent (Life Technologies) between a microscope glass slide and an N1.5 glass coverslip. The mounted samples were stored at room temperature and protected from light for 12 hours to allow the ProLong reagent to cure. Prints were imaged using a Leica SP5 multiphoton microscope with a 10× (NA = 0.4) objective and a 25× (NA = 0.95) water immersion objective. 3D image stacks were deconvolved with AutoQuant X3 and processed with Imaris 7.5.

### Perfusion of 3D printed coronary arterial tree

To evaluate whether the 3D printed arterial tree was manifold, we mounted it in a custom-made 3D printed perfusion fixture (fig. S7, A and B). A solution of 11 mM CaCl_2_ (Sigma) and 0.1% (w/v) black food coloring was injected into the root of the tree using a standard 3-ml syringe (BD Biosciences) with a 150-μm-ID, 0.5-inch needle, and the tip at the end of each branch was cut off to permit outflow. Perfusion was captured with a digital camera (D7000 SLR, Nikon) mounted on a stereomicroscope with oblique illumination (SMZ1000, Nikon).

### Creation of a 3D model of the heart of a 5-day-old chick embryo

The 3D model of the embryonic chick heart was generated from 3D optical imaging data of a fluorescently labeled 5-day-old heart. Fertilized eggs of White Leghorn chicken were incubated at 37°C and 50% humidity for 5 days to do this. Then, the embryo [Hamburger-Hamilton stage 27 to 28 (*39*)] was explanted and the heart (ventricles, atria, and outflow tract) was dissected and fixed for 15 min in PBS with calcium, magnesium, and 4% formaldehyde. After being washed in PBS, the heart was blocked and permeabilized for 2 hours at 37°C in PBS with 0.1% Triton X-100 and 5% goat serum. Two steps of immunostaining were carried out overnight at 4°C. The first stain used dilutions of 1:200 DAPI, 3:100 phalloidin conjugated to Alexa Fluor 633 (Life Technologies), and 1:100 anti-fibronectin primary antibody (mouse, Sigma-Aldrich). After being extensively washed in PBS, the samples were stained with a 1:100 dilution of goat anti-mouse secondary antibody conjugated to Alexa Fluor 546 (Life Technologies). Samples were then washed and dehydrated by immersion in successive solutions of PBS with an increasing concentration of isopropyl alcohol as previously described (*40*). Finally, the samples were cleared by transferring to a solution of 1:2 benzyl alcohol/benzyl benzoate (BABB) to match the refractive index of the tissue. The transparent sample was mounted in BABB and imaged with a Nikon AZ-C2 macro confocal microscope with a 5× objective (NA = 0.45).

The 3D image stack was deconvolved using AutoQuant X3 and processed with Imaris 7.5, MATLAB (MathWorks), and ImageJ. The DAPI (fig. S8A), actin (fig. S8B), and fibronectin (fig. S8C) channels were merged to obtain an image with the simultaneously well-defined trabeculae and outer wall of the heart (fig. S6D). A detailed mask of the heart showing the trabeculae was created by segmenting the averaged signals using a high-pass threshold (fig. S8E). A rough mask showing the bulk of the heart was obtained using a low-pass threshold (fig. S8F). Next, the Imaris “Distance Transform” XTension was used on the bulk mask to create a closed shell of the outer wall of the heart. The high-detail mask and the mask of the closed shell were combined to obtain a complex model of the heart with detailed trabeculae and a completely closed outer wall (fig. S6G). The final model was smoothed and segmented using Imaris to preserve a level of detail adequate for 3D printing (fig. S8H). A 3D solid object was created by exporting the smoothed model as an STL file using the Imaris XT module and the “Surfaces to STL” Xtension for MATLAB (fig. S8, I and J).

### Mechanical characterization

Mechanical characterization comparing 3D printed and cast alginate constructs was performed using uniaxial tensile testing, adapted from our previously published method for characterizing soft PDMS (*41*). Briefly, tensile bar strips (dog bones) of 4% (w/v) alginic acid in 11 mM CaCl_2_ were either 3D printed using the FRESH method or cast into laser-cut acrylic molds consisting of a grip section (7 × 10 mm), a reduced section (3.45 × 25 mm), and ~1 mm thickness. The 3D printed strips were fabricated with a 250-μm-diameter nozzle in a slurry containing 11 mM CaCl_2_. Settings for the 3D printed strips were 100-μm layers, 50% octagonal infill, and 1 perimeter. The width and thickness of each test strip were individually measured before mechanical analysis. Uniaxial tensile testing (*n* = 6 of each type) was performed on an Instron 5943 (Instron) at a strain rate of 5 mm/min until failure. The elastic modulus of each sample was determined from the slope of the linear region of the stress-strain curves from 5 to 20% (or until failure, if it failed before 20%).

## SUPPLEMENTARY MATERIALS

Supplementary material for this article is available at http://advances.sciencemag.org/cgi/content/full/1/9/e1500758/DC1 Fig. S1. Modification of an open-source 3D printer for FRESH printing. Fig. S2. Preparation of the gelatin slurry support bath. Fig. S3. Examples of 3D printed bifurcated tubes using alginate, fibrin, and collagen. Fig. S4. 3D printed sheets of cells and ECM. Fig. S5. Mechanical characterization of cast and 3D printed alginate dog bones using uniaxial tensile testing. Fig. S6. A comparison of the 3D model and 3D printed arterial tree to assess print fidelity. Fig. S7. A 3D printed perfusion fixture for the right coronary arterial tree. Fig. S8. Generation of a 3D model of the embryonic heart from confocal microscopy. Fig. S9. A comparison of the 3D model and 3D printed embryonic heart to assess print fidelity. Fig. S10. A comparison of the 3D model and 3D printed brain. Movie S1. Time lapse of FRESH printing and heated release of the “CMU” logo. Movie S2. FRESH printing using the dual syringe pump extruders. Movie S3. Out-of-plane FRESH printing of a helix. Movie S4. Uniaxial strain of a FRESH printed femur model showing elastic recovery. Movie S5. Strain to failure of a FRESH printed femur. Movie S6. FRESH printing of soft collagen type I constructs. Movie S7. Time-lapse video of a coronary arterial tree being FRESH printed. Movie S8. Perfusion of a FRESH printed coronary arterial tree. Movie S9. Visualization of the 3D structure of a FRESH printed brain model. Movie S10. Modification of a sub-$400 3D printer for FRESH printing.
